# A Brief Guide to the Structure of High-Temperature Molten Salts and Key Aspects Making Them Different from Their Low-Temperature Relatives, the Ionic Liquids

**DOI:** 10.1021/acs.jpcb.1c01065

**Published:** 2021-05-28

**Authors:** Shobha Sharma, Alexander S. Ivanov, Claudio J. Margulis

**Affiliations:** † Department of Chemistry, The University of Iowa, Iowa City, Iowa 52242, United States; ‡ Chemical Sciences Division, Oak Ridge National Laboratory, 1 Bethel Valley Road, Oak Ridge, Tennessee 37830, United States

## Abstract

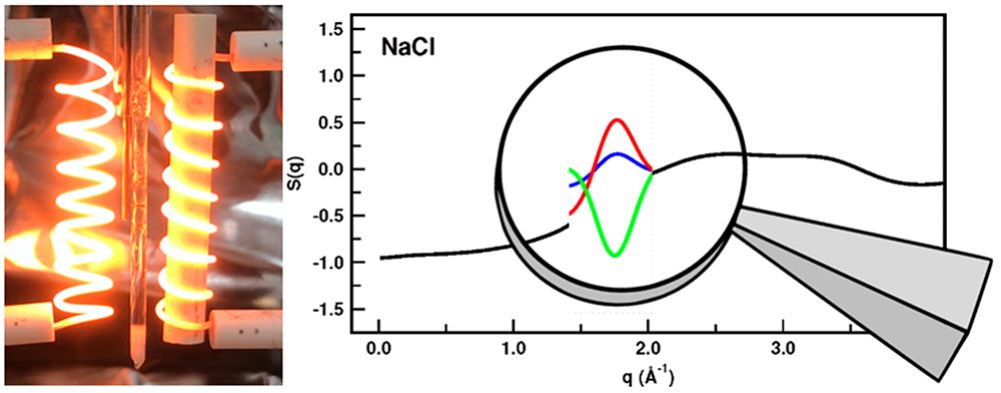

High-temperature molten salt research is undergoing somewhat of a renaissance these days due to the apparent advantage of these systems in areas related to clean and sustainable energy harvesting and transfer. In many ways, this is a mature field with decades if not already a century of outstanding work devoted to it. Yet, much of this work was done with pioneering experimental and computational setups that lack the current day capabilities of synchrotrons and high-performance-computing systems resulting in deeply entrenched results in the literature that when carefully inspected may require revision. Yet, in other cases, access to isotopically substituted ions make those pioneering studies very unique and prohibitively expensive to carry out nowadays. There are many review articles on molten salts, some of them cited in this perspective, that are simply outstanding and we dare not try to outdo those. Instead, having worked for almost a couple of decades already on their low-temperature relatives, the ionic liquids, this is the perspective article that some of the authors would have wanted to read when embarking on their research journey on high-temperature molten salts. We hope that this will serve as a simple guide to those expanding from research on ionic liquids to molten salts and *vice versa*, particularly, when looking into their bulk structural features. The article does not aim at being comprehensive but instead focuses on selected topics such as short- and intermediate-range order, the constraints on force field requirements, and other details that make the high- and low-temperature ionic melts in some ways similar but in others diametrically opposite.

## Introduction

There has been a constant stream of articles^1−69^ including salts in their molten or glass states since the early 1900s; see for example, the works of Zachariasen^70^ and Rosenhain.^71^ Yet, through the years, molten salts continue to be rediscovered for applications in energy technologies including nuclear energy,^72−76^ solar energy harvesting,^77−79^ batteries,^80−82^ and separations^83^ to mention just a few. The 1970s and early 1980s brought a series of interesting spectroscopic measurements that could be directly linked to the 3D structure of salts in the molten phase, in particular via Raman spectroscopy,^16−18,22,23,29^ and soon after, pioneering X-ray and neutron scattering results^30,31,33−36,38,43^ revealed more information about short and intermediate range order. These results followed or were followed by pioneering theory developments and early simulation work.^11−13,15,19−21,24−28,32,37,39,41,42,44−52,59^ Multiple force fields to simulate salts have been developed, but modern point-polarizable force fields with quantum mechanical accuracy^53−58,61^ are the current go-to models for most simulations of divalent and multivalent cationic systems coupled with polarizable anions. Recently, a faster Drude-type model that includes some of the same ingredients as in these other more expensive models has also been successfully developed and applied to the case of MgCl_2_ and its mixtures with KCl.^84^ A variety of articles based on machine-learning simulation schemes for molten salts have also recently appeared in the literature.^85−90^ Advances in computational models have come hand-in-hand with new experimental studies,^40,59,60,62−69,91−94^ and in some cases, the combination of these challenges strongly entrenched assumptions, for example the octahedral coordination of U^3+^,^94^ the tetrahedrality in coordination for Mg^2+^,^58,95^ and other properties that collectively have influenced the literature for 30 years or more.^58,93−96^ The works cited thus far are meant to provide some context and are not exhaustive or cover many of the most studied salts and their mixtures of which the eutectics are of special relevance.

**Figure 1:**
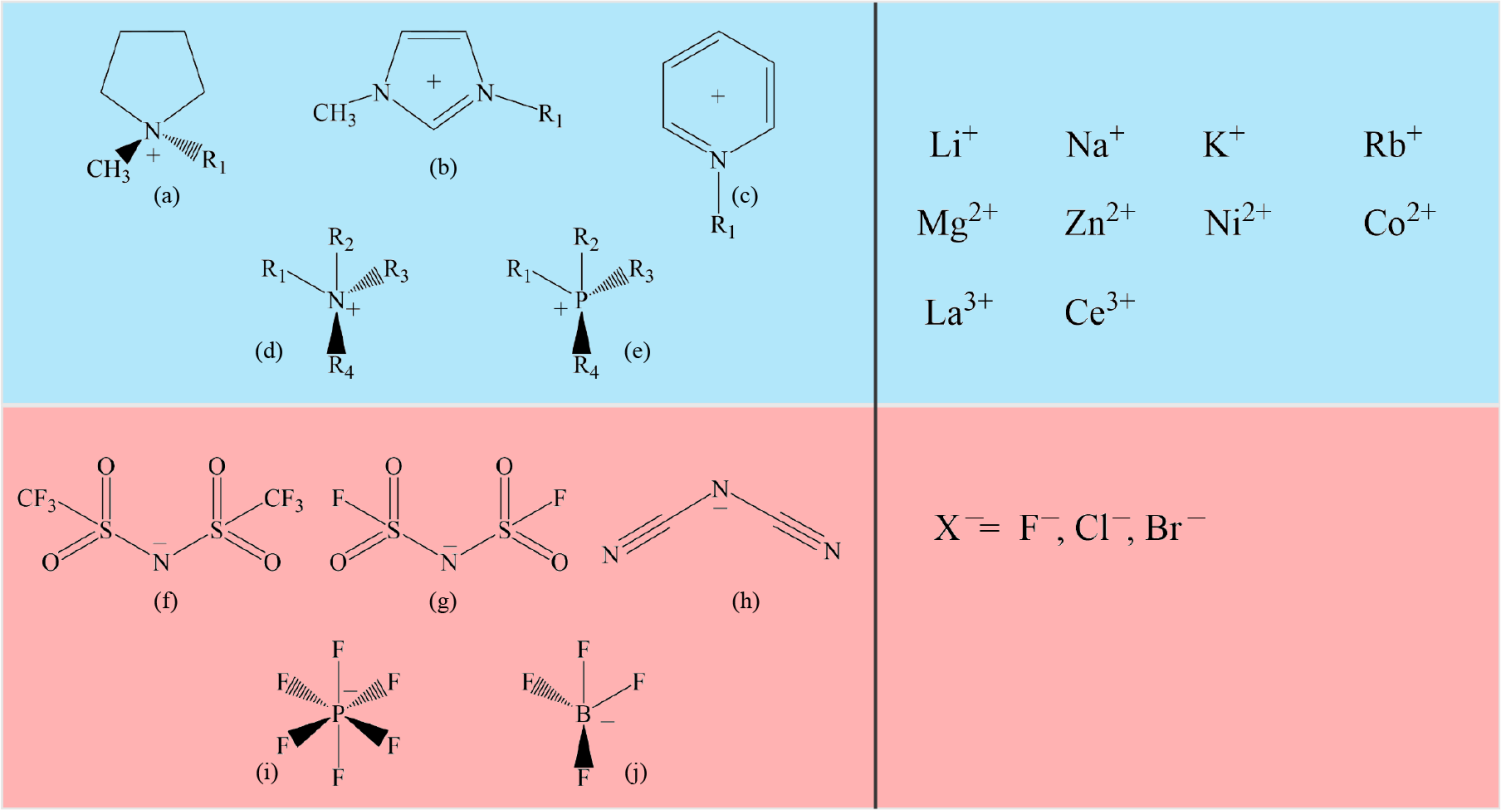
(Left) some common ions or ion families associated in the literature with ionic liquids and (right) typical ions associated with high-temperature molten salts.

Literature on ionic liquids (ILs) also dates to the early 1900s with the work by Walden^97^ on ethylamonium nitrate, and a few decades later to work on chloroaluminates.^98^ The reader is encouraged to read the authoritative history of ionic liquids recently published by Welton and references therein.^99^ For the purpose of this perspective article, when comparisons are made between molten salts and ILs (see Figure 1), these are made with the most common modern ILs in mind, which are based on organic cations and organic or inorganic anions instead of the early chloroaluminates.

## Results and Discussion

Before we embark into a detailed discussion about X-ray and neutron scattering, including the temperature dependence of the features in the structure factor, it is worth highlighting some experimental and computational peculiarities associated with these systems that will be useful to those coming from more conventional condensed phase liquid systems.

### Experimental Caveats and Considerations

Over the past decades, advancements in high-energy X-ray and enhanced flux neutron diffraction techniques have provided opportunities to obtain the structure function for liquid salts out to large momentum transfers (*q*), leading in principle to high-resolution pair distribution functions that can be directly compared to simulation results and thus are important as a benchmark for the development of accurate atomistic models. Confidence in such models is important for the prediction of transport and thermodynamic properties, as some of these are challenging to measure for salts at high temperature. An example is ionic diffusivity, which is readily accessible for ILs via NMR measurements but less so for molten salts. The principles of X-ray/neutron diffraction theory and the details of total scattering data processing are extensively discussed elsewhere^100−104^ and need not be elaborated here, except perhaps to mention that notation and nomenclature in the literature have taken slightly different forms for molten salts and ILs. In fact, even within the molten salts literature, there is significant variation in the use of variables; an excellent resource to start understanding these is a comprehensive review by Keen.^105^

**Figure 2:**
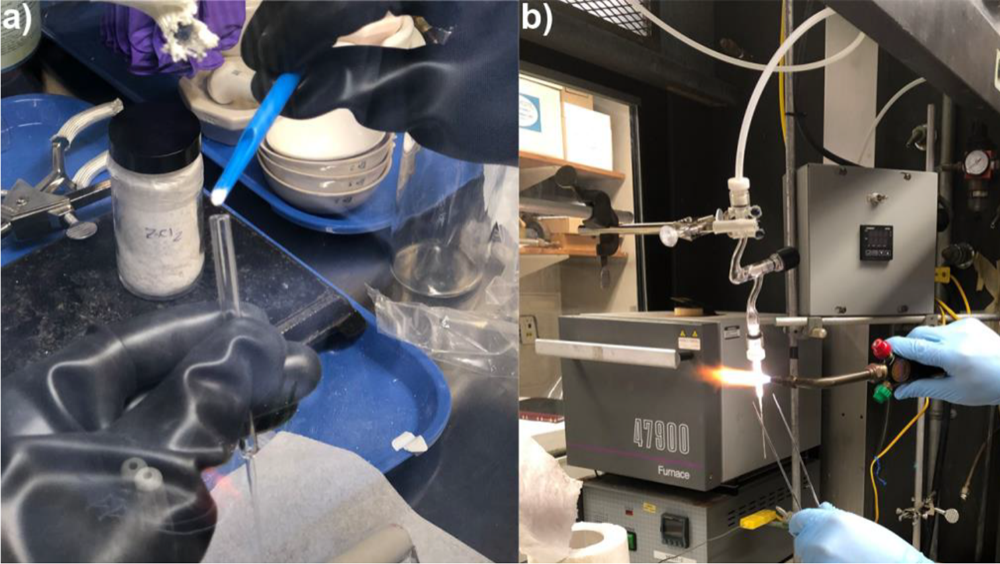
(a) Loading the salt sample in a glovebox. (b) Sealing the sample in a capillary.

**Figure 3:**
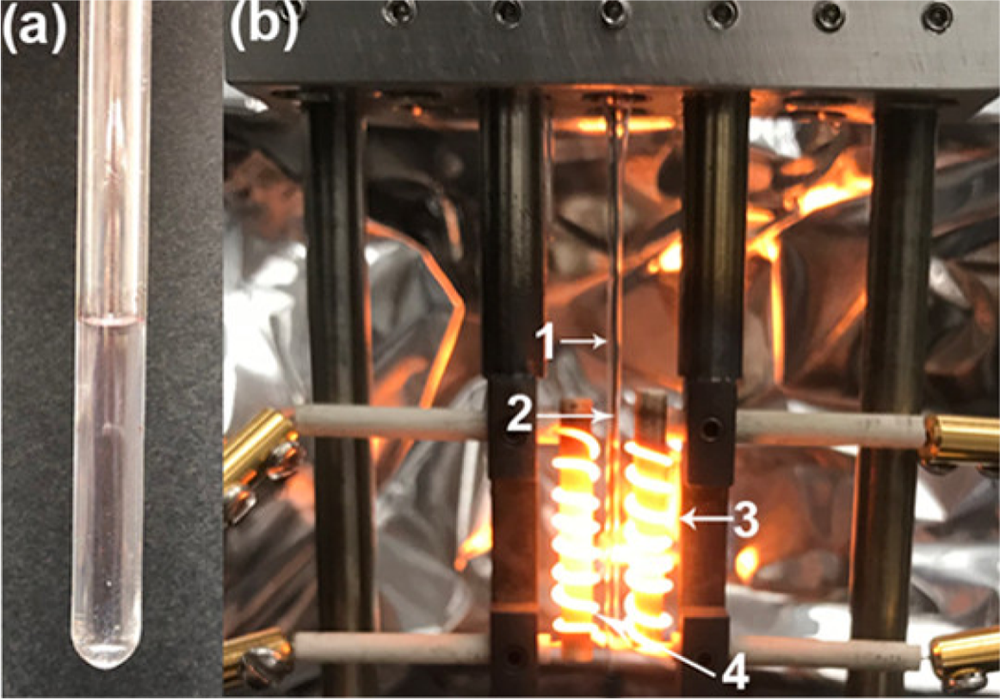
(a) Inorganic molten salt mixture (MgCl_2_/KCl) at 1073 K. (b) Experimental setup typically used for high-energy X-ray scattering measurements: 1, quartz tube; 2, thermocouple; 3, heater; 4, salt sample. Reprinted with permission from ref (61). Copyright 2020 American Chemical Society.

Total X-ray and neutron scattering measurements are not without some experimental uncertainty stemming from a number of challenges posed not only by the chemistry of the particular salt but also by the extreme conditions under which such measurements are carried out. In order to obtain reliable X-ray or neutron scattering data, one needs first to make sure that samples are of high quality and their compositions accurately known. Due to the hygroscopic nature of ILs and molten salts, this constitutes a challenge. Whereas the problem is well understood and relatively easy to control in the case of ILs, it is significantly more challenging for molten salts where the quick uptake of atmospheric water for some very hygroscopic salts may cause noticeable changes in sample composition, leading to uncertainties in experimental data reduction and analysis. Some inorganic salt hydrates readily undergo hydrolysis upon a temperature increase, resulting in a variety of unanticipated chemical species such as metal oxides.^106^ Therefore, moisture-sensitive samples are usually prepared in an inert atmosphere glovebox and loaded into 1–2 mm quartz capillaries. While IL samples are usually sealed with a silicon rubber or wax to prevent water absorption, inorganic salt samples need to be flame-sealed to enable X-ray/neutron diffraction data collection at high-temperature regimes (see Figure 2). In addition, when compared with ILs, a more complex measurement setup is needed for molten salts where the quartz tube is placed in a furnace sample holder consisting of two resistive heaters as shown in Figure 3. Note, however, that quartz is by no means the universal containment material for all possible salts or salt mixtures, since for example fluorides tend to react with glass at elevated temperatures, requiring the utilization of Pt–Rh^107^ or boron nitride containers in the case of X-ray scattering measurements.

In addition to the described chemical and technical complexities, there is also the fact that the structure function (*S*(*q*)) and the pair distribution function (PDF) obtained via Fourier transformation of *S*(*q*) are not quantities that are directly measured but instead are derived from experimental measurements of scattering intensity. Uncertainties or errors in *S*(*q*) are often due to inaccuracies in intensity counting and approximations applied in data treatment. Specifically for molten salts, the rapid decay of the scattering intensity with increasing *q*, the low signal-to-background ratios (the total scattering signal for molten salt can be swamped by the scattering contributions of furnaces and containers) and difficulties in obtaining the structure factor to sufficiently high *q*-values can lead to problems in the determination of absolute normalizations and the subsequent derivation of high-resolution PDFs.

**Figure 4:**
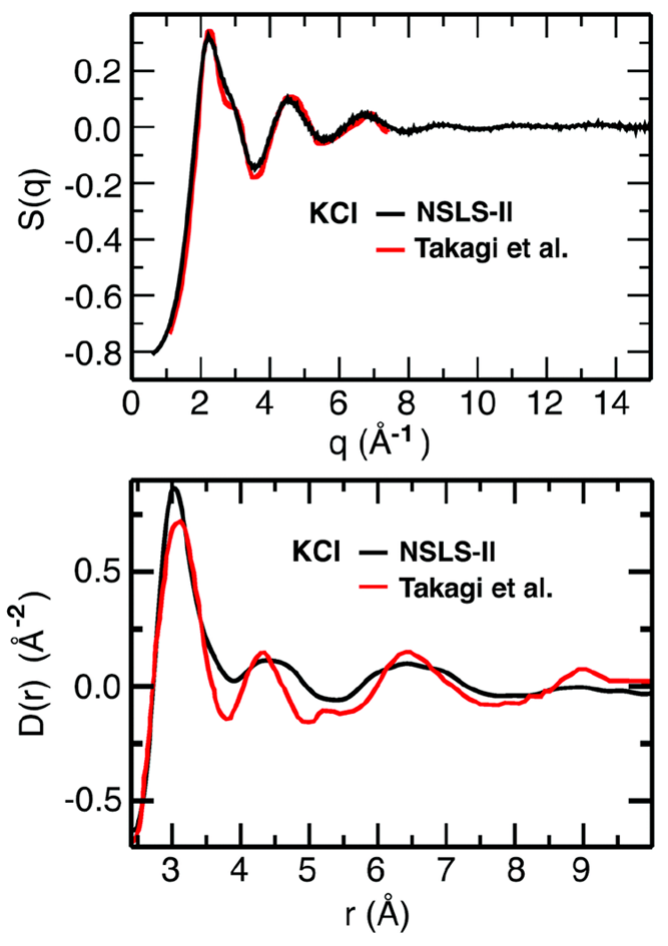
(Top) structure function, *S*(*q*), recently obtained by our group at NSLS-II contrasted with that of Takagi et al.^28^ (Bottom) Real space *D*(*r*) functions derived from these. Reproduced from ref (95) with permission from the PCCP Owner Societies.

It is unfortunately not uncommon to see discrepancies in peak intensities between the X-ray/neutron *S*(*q*) and PDF results reported by different research groups for a given molten salt system. These experimental inconsistences may cause uncertainty in the selection of the most reliable first-principles or classical force field methodology to interpret scattering and spectroscopic results from an atomistic perspective. To provide the most accurate experimental *S*(*q*) values or PDFs, a wide range of experimental data for the same system and conditions would be desirable, including from different synchrotron/neutron sources, but such checks are scarce. In addition, direct *S*(*q*)/PDF comparisons with pioneering work in the literature are often also hindered by specific data treatments and processing protocols, e.g., different *S*(*q*) normalization, the definition of the composition unit, and the use of modification functions. A particular sticking point is that pioneering work on molten salts was done using conventional X-ray and low flux neutron sources, leading to *S*(*q*) functions with quite limited q-range and significant statistical errors. To highlight this point, Figure 4 compares our recent high-energy X-ray (74.4 keV) scattering results^95^ for molten KCl obtained at the National Synchrotron Light Source II (NSLS-II) with those reported by Takagi et al.^28^ using Cu Kα (8.0 keV) radiation. One may see noticeable discrepancies in the real-space PDF, *D*(*r*), especially with respect to the positions and intensities of peaks associated with short-range order. These differences are crucially important when trying to understand coordination numbers and solvation environments for the ions. The observed inconsistencies are primarily due to termination errors at large *q* in Takagi’s *S*(*q*) data reported only up to *q* = 7.38 Å^–1^—the highest momentum transfer attainable by the Cu Kα source in the range of the considered scattering angles.^28^ Although the *q*-range for this earlier study is limited, the agreement with modern synchrotron measurements in the first three peaks of *S*(*q*) is quite good, and this is particularly encouraging, since the structure functions were obtained from different instruments using different modes (reflection vs transmission), and as a consequence, they were derived through treatments in which various corrections had to be performed in different ways. The fact that, at least on a restricted *q*-range, pioneering results and data collected on more advanced synchrotron beamlines overlap provides confidence in the possibility to collect highly reproducible experimental data for molten salts which will serve as excellent benchmarks for theoretical models.

### Computational Caveats and Considerations

Besides the obviously different temperature regimes on which measurements on IL and molten salt systems are carried out, from a computational perspective, a few other notable issues should be highlighted. In general, the lowest room-temperature IL viscosities are on the order of 10–20 times that of liquid water at atmospheric conditions, whereas other ILs have much higher viscosities that can be hundreds of cP or more.^108−110^ Instead, high-temperature molten salts have low viscosities often on the order of 1 cP or sometimes less.^111,112^ What this means is that from a computational perspective, guaranteed convergence of properties requires careful consideration in the case of ILs (simply put, simulations may require tens or hundreds of nanoseconds to converge depending on the property and temperature).^113,114^ The issue of the high viscosity of ILs is compounded by the fact that force fields, particularly nonpolarizable force fields or those in which charges have not been scaled to account for some level of polarization or charge transfer, have actual model viscosities that are sometimes orders of magnitude larger than the experimental ones.^108,115^ In other words, whereas an IL may be viscous but not in the glass regime, its fixed-charge force field model version may well be.

**Figure 5:**
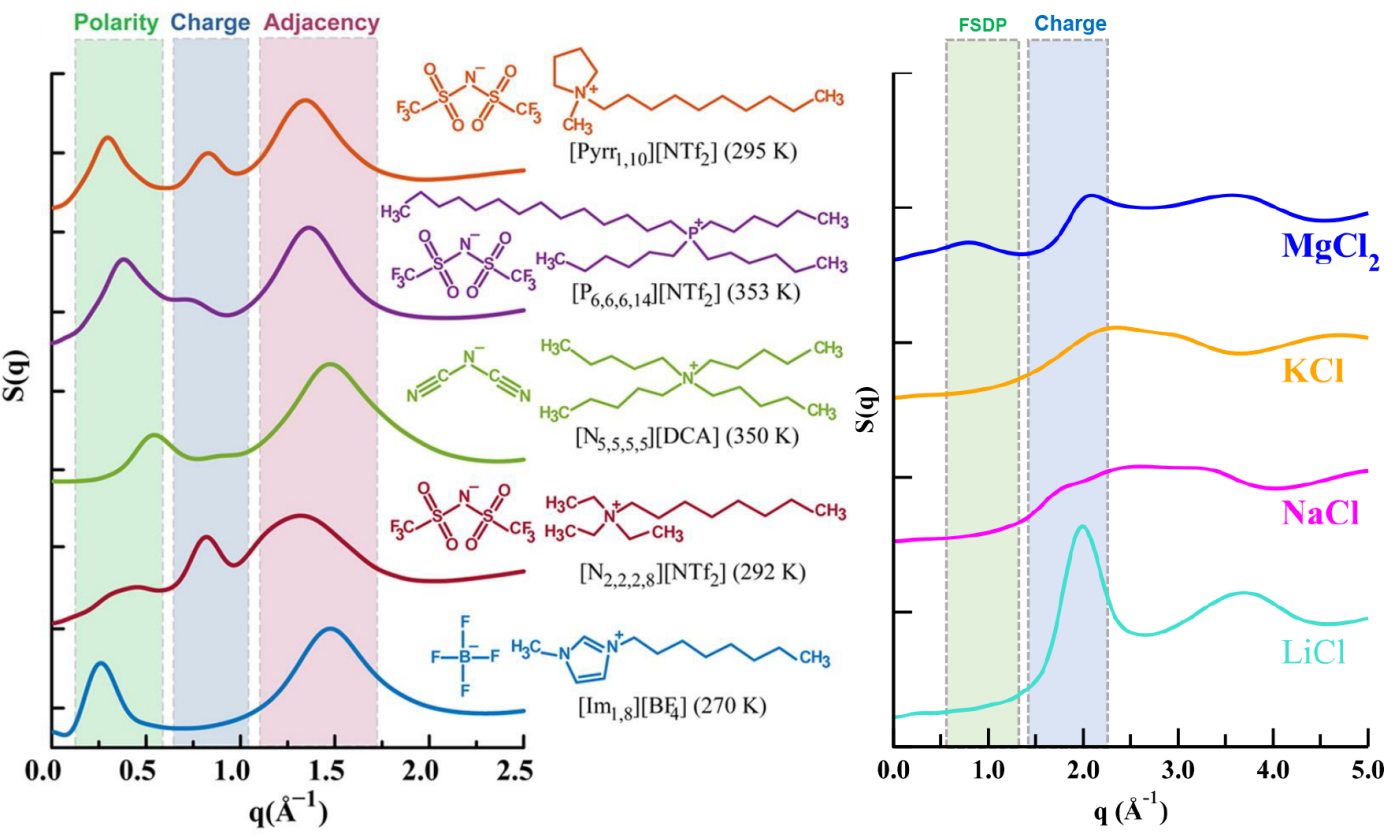
(Left) for different ILs, *S*(*q*) highlighting regimes associated with polar–apolar alternation (this is the prepeak or first sharp diffraction peak), with charge alternation, and with vicinal adjacency correlations. (Right) for different molten salts, *S*(*q*) highlighting the charge alternation region and when present the prepeak. Notice that charge alternation and prepeak regions occur at significantly higher *q* values for molten salts. Left panel reprinted with permission from ref (119). Copyright 2015 American Chemical Society.

*Yet, from a practical perspective particularly when interested in the structure function, models for ILs are much more forgiving than those for molten salts.* What we mean by this is that whereas it is easy to get transport properties wrong (sometimes very wrong) for ILs, it is hard for a reasonable force field to not show the three typical characteristics of an IL: which are polar–apolar alternation [the so-called prepeak or first sharp diffraction peak (FSDP)], charge alternation, and adjacency correlations.^116−126^ Each of these features appears in a specific *q*-range, as shown in Figure 5 (left).^119,127−134^ Instead, structural properties of molten salts including coordination numbers and intermediate range order appear to be quite sensitive to the model and, as opposed to the case for ILs, it is very easy to get these wrong. Because of this, it is often the case that the Coulomb and dispersion-repulsion energy functions used for molten salts are more complex than those commonly used for ILs.^135^ This is true even for the more complex force fields for ILs based on the polarizable Drude model.^115,136−142^ The issue is most problematic when coupling small multivalent cations such as Mg^2+^ with highly polarizable anions such as Cl^–^.^84,143^ Because of the very short distance between these ions in the condensed phase, the physical validity of the commonly used point-induced dipole approximation within the polarizable ion model (PIM) requires careful consideration; this is less of an issue for ILs where interionic distances are larger and formal charges smeared over multiple atoms. To avoid unphysical overpolarization in the case of molten salts and to get structural and dynamical properties right, the key ingredient is the collection of pairwise damping functions used to correct the charge-induced dipole interactions.^53−58,61,84^ This often results in force field parameters that are only good to simulate a specific system but are not easily transferable. In other words, for molten salts with multivalent cations and polarizable anions, do not expect to find parameters for a given ion in the literature and assume that those can be trivially combined with other parameters even when the force field has the same functional form. Because of their low viscosity, the number of nanoseconds in a molecular dynamics simulation run needed to converge molten salts properties are in general lower than for ILs but actual wall times tend to be comparable or even longer due to the complexity of force fields.^84^

### Why Are Features in *S*(*q*), Particularly Those Associated with the FSDP, Much More Sensitive to the Force Field in the Case of Inorganic Molten Salts?

Let us consider a prototypical ionic liquid such as [C_*n*_mim][PF_6_], below *n* = 4 the first sharp diffraction peak is not prominent or observed and for *n* = 6, 8, 16 the prepeak is at *q* = 0.350, 0.297, and 0.183 Å^–1^ respectively.^144^ How bad would a force field need to be in order to produce a prepeak that is off by about 0.1 Å^–1^ in the case of [C_16_mim][PF_6_]? A trivial estimation for the periodicity of the prepeak is 2π/0.183 ≈ 34.33 Å; a shift to the left by 0.1 Å^–1^ would result in 2π/0.083 ≈ 75.70 Å. This is about 40 Å off in real space! Instead, if we consider MgCl_2_ where the prepeak appears at 0.85 Å^–1^,^58^ the same shift in *q* would result in a real space distance difference of about 1 Å; 2π/0.85 ≈ 7.39 Å whereas 2π/0.75 ≈ 8.38 Å. In other words, most reasonable force fields for ILs will get the prepeak position correctly, but instead, small changes in a force field can have major consequences on the shape and position of the features in *S*(*q*) for a typical molten salt.

### The Defining Feature for Molten Salts Is the Two Peaks and One Antipeak Associated With Charge Alternation

Many times but not always, a peak in the overall *S*(*q*) corresponding to charge alternation is prominent and called “the main peak” in the literature.^145,146^ However, in other cases this peak is completely absent and the main peak has a different structural origin. This is true both for molten salts and for ILs. We see this clearly from Figure 5 (left) where [Im_1,8_][BF_4_] shows no peak in the charge alternation region around 0.8 Å^–1^ but [N_2,2,2,8_][NTf_2_] shows a prominent peak; we also see this from Figure 5 (right) where for KCl there is no sign of a peak around 1.8 Å^–1^ but LiCl shows a prominent peak just below 2 Å^–1^. The key point is that all molten salts and ILs are defined by charge alternation, but *whether the charge alternation peak is prominent or completely absent is simply a matter of contrast in the technique*.^116^

**Figure 6:**
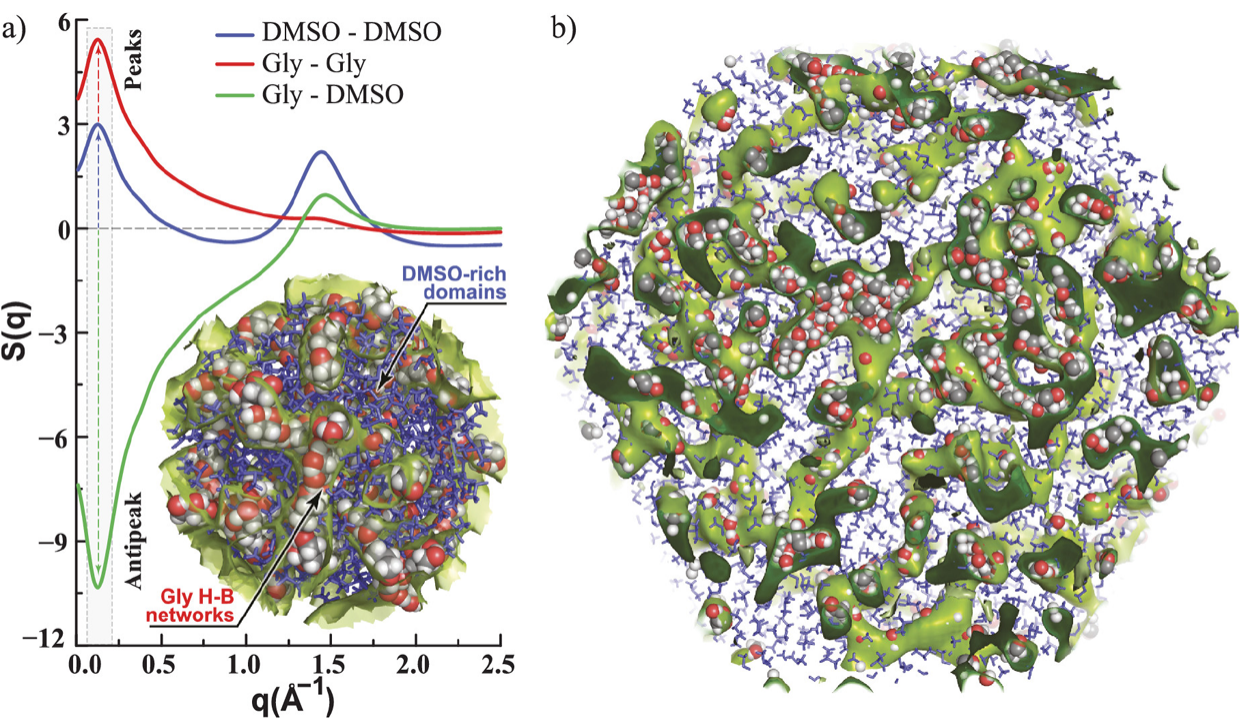
(Left) partial subcomponents of *S*(*q*) for a mixture of the conventional solvents dimethyl sulfoxide and glycerol. The figure highlights the two peaks and one antipeak associated with the pattern of alternation associated with the distance between glycerol chains spaced by dimethyl sulfoxide domains; (right) a larger view of the system highlighting these alternations in real space.^147^ Reprinted with permission from ref (147). Copyright 2015 American Chemical Society.

We begin to understand this when we notice that for any liquid, alternations at any particular length scale are associated with two peaks and one antipeak in the partial subcomponents of *S*(*q*) in the same *q* region.^116,119^ For example, a mixture of glycerol and DMSO can become nanoheterogeneous with nanoscopic chains of glycerol spaced by DMSO domains, as can be seen in Figure 6. The typical distance between two glycerol chains is about the same as the typical distance between DMSO domains intercalated by a glycerol chain. This results in a peak at low *q* values for the DMSO–DMSO subcomponent of *S*(*q*) and another peak in the same region for the glycerol–glycerol subcomponent. Because the two patterns of alternation in the case of glycerol and DMSO are shifted by half a period (DMSO domains are intercalated by glycerol chains and glycerol chains are separated by DMSO domains) the DMSO–glycerol subcomponent of *S*(*q*) appears as an antipeak or a negative going peak at the same *q* region. The concept is the same for any other type of alternation and certainly applies to the charge alternation behavior of molten salts and ILs albeit at a larger *q* value.^116^

**Figure 7:**
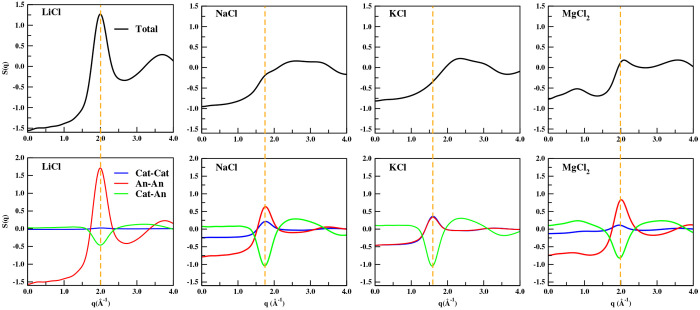
For LiCl, NaCl, KCl, and MgCl_2_, (top) total *S*(*q*) and (bottom) partial subcomponents of *S*(*q*). A vertical dashed line highlights how the sum of two peaks and one antipeak in some cases results in a peak in the overal *S*(*q*) but in others there is a shoulder or even no noticeable feature due to cancellations. Data for LiCl is at 1148 K, KCl at 1173 K, and MgCl_2_ at 1073 K.^61,95^

Whether these peaks and antipeaks are prominent in the subcomponents of *S*(*q*) depends on the corresponding values of the X-ray atomic form factors or neutron scattering lengths. This is clearly depicted in Figure 7 where for KCl the formal number of electrons for K^+^ and Cl^–^ are the same and the ionic sizes are very similar, hence the *q*-dependent form factors are also quite similar. The two peaks (K–K and Cl–Cl) superpose, and the K–Cl antipeak completely cancels them.^95^ This results in large partial subcomponents of the structure factor completely canceling out to yield an X-ray *S*(*q*) that is featureless in the charge alternation regime! The opposite is true for LiCl where there is significant difference in the size and number of electrons of the species creating contrast between the anion and the cation that translates into an easily observable charge alternation peak in the overall *S*(*q*). The cases of NaCl and MgCl_2_ are intermediate between these two where the contrast is reasonable in the case of MgCl_2_ and a prominent peak is observed but only a shoulder appears in the overall *S*(*q*) for NaCl. Because neutron scattering and X-ray scattering depend on the details of the nuclear structure of the species in one case and the electronic structure of the species in the other, a charge alternation peak that is completely absent in the X-ray *S*(*q*) can be prominent in the neutron *S*(*q*). In conclusion, all molten salts are characterized by charge alternation in the subcomponents of *S*(*q*), but only in some cases these do not cancel and give rise to a prominent main charge alternation peak in the overall X-ray or neutron *S*(*q*). It is not safe to assume that the main peak is always associated with charge alternation, but it is safe to assume that if a charge alternation peak is not present in *S*(*q*), this is simply due to a technique limitation and not due to lack of charge alternation in the condensed phase.

How are ionic liquids different or similar to molten salts when thinking about charge alternation? When we think of molten salts, we envision single-atom cations and anions that alternate in a 3D pattern linked to their relative sizes, polarizabilities, formal charges, etc. Instead, in the case of prototypical ILs, it is often the cation that has a “polar head” and an “apolar tail”, and it is the cation polar head and not the full cation that alternates with the anions, which are often but not always symmetrical and without an apolar component^117,121,122,125,126^ (some anions are asymmetric and can have extensive fluorinated or other type of tails, but this is not the subject of our discussion). It is then the cation head–cation head and the anion–anion subcomponents of *S*(*q*) that manifest as peaks in the charge alternation reciprocal space region and the cation head–anion subcomponents that show as an antipeak. Notice that this charge alternation region in reciprocal space is at significantly different q values for ILs and molten salts (≈0.8 Å^–1^ in Figure 5 (left) and ≈1.8 Å^–1^ in Figure 5 (right), respectively). We emphasize again that it is easier for force fields of ionic liquids to capture this feature correctly as it appears at significantly smaller *q* values (larger distances). All other considerations are the same as described in the paragraph above. All ionic liquids and molten salts display charge alternation, but only in some cases this shows in the overall neutron or X-ray scattering *S*(*q*) due to contrast.^121^

### Intermediate Range Order for Molten Salts

**Figure 8:**
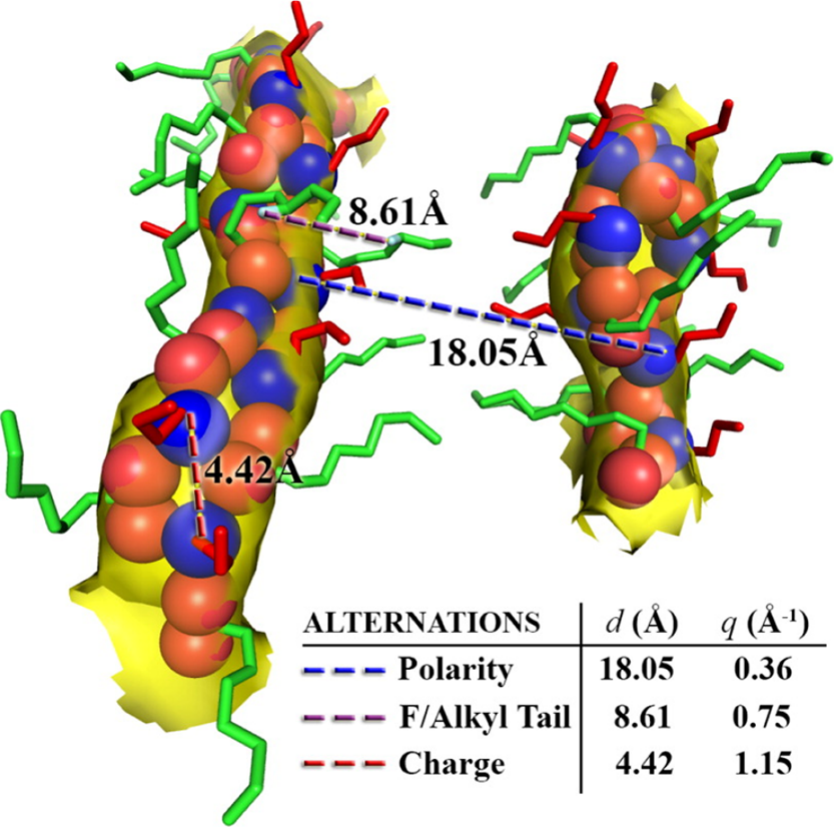
For an ionic liquid, two charge networks spaced by tails.^122^ Reprinted with permission from ref (122). Copyright 2014 American Chemical Society.

In the early 2010s, there was significant controversy regarding the origin of the prepeak in ILs.^117,144,148−150^ In hindsight, and for prototypical ILs, this problem is very simple and has been fully resolved.^116,119,129,144,151−186^ For ILs, where cations have a charged head and a significantly large apolar tail, the prepeak is due to the typical distance between charge networks alternated by apolar regions, or apolar domains alternated by the charge network.^116−126^ Since this is a pattern of alternation, same type species (polar–polar and apolar–apolar) show peaks in partial subcomponents of *S*(*q*) at the prepeak region, whereas opposite type species (polar–apolar) show an antipeak; notice that positive and negative species are considered “same type species” in this *q* regime because they are both polar.^123,124^ This is clearly depicted in Figure 8, where polar cationic and anionic heads form networks that are separated by other networks via tails that act as spacers.

Pinpointing the origin of intermediate range order in the case of molten salts is significantly more difficult. Molten salts do not have apolar regions, and the prepeak does not necessarily stem from a simple pattern of alternation with two peaks and an antipeak in the partial subcomponents of *S*(*q*) as in the case for ILs. The reader can immediately see the absence of this pattern from the subcomponents of *S*(*q*) for MgCl_2_ in Figure 7 at about 0.85 Å^–1^.

So what gives rise to a prepeak in molten salts? This has been studied extensively and a particularly good discussion can be found in a review article by Wilson.^187^ Our group has studied the case of MgCl_2_ and its mixtures with KCl and what we found is that MgCl_2_ is a networked liquid with chains of Cl^–^-decorated Mg^2+^ ions.^58,61,84^ These in-network Mg^2+^ ions share Cl^–^ counterions, but there are nearby Mg^2+^ ions that are either part of a different network or simply form a cluster and do not share Cl^–^ counterions with the first network. The distance between Mg^2+^ ions sharing counterions is different from that of those not sharing counterions. The first distance, which is shorter, has to do with the charge alternation feature in *S*(*q*), whereas the second has to do with the prepeak.^61^ In other words, just like in the case of ILs, the prepeak has to do with the distance between charge networks. In the case of ILs these charge networks are spaced by tail domains, but in the case of MgCl_2_ there is no spacer. Whereas these two characteristic distances in the case of MgCl_2_ are different, the difference is not very large when contrasted with the difference between typical charge alternation vs polarity alternation distances for ILs. Being able to resolve these differences computationally in the case of molten salts depends on the intricacies of the polarizable force field and damping functions. In general, our experience is that nonpolarizable force fields or core–shell type models are unable to do so but our recently developed SEM-Drude model which uses charge-dipole damping functions akin to those in the more expensive PIM can. Perhaps, one could come up with a clever way to partition and label ion collections to see the prepeak of molten salts as an alternation pattern. However, this would be computationally impractical because such labels would necessarily change from simulation frame to simulation frame, and as opposed to the case for ILs, they would be ion location-dependent instead of ion type-dependent.

**Figure 9:**
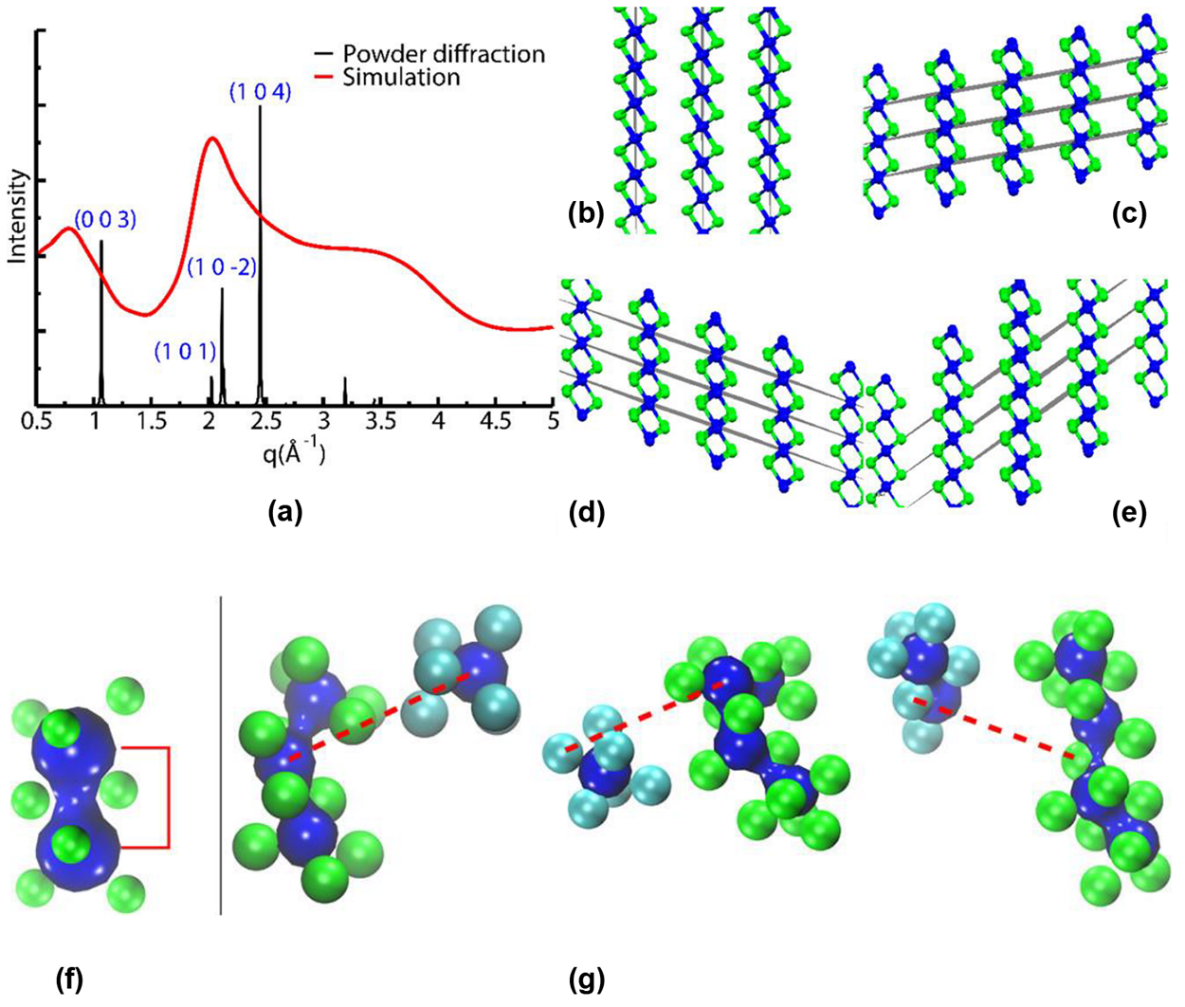
(a) Powder diffraction peaks and liquid phase intensity for MgCl_2_. (b) (003), (c) (102̅), (d) (101), and (e) (104) Bragg planes in the MgCl_2_ crystal. Planes (003) exemplify across network prepeak behavior, (102̅) and (101) planes charge alternation behavior, and (104) planes are one of the many possible examples of adjacency correlations. (f) Depicted as a surface two Mg^2+^ ions sharing at their waist Cl^–^ counterions (charge alternation behavior). (g) Examples of across network interactions (prepeak behavior). Top figure reprinted with permission from ref (58) and bottom figure reprinted with permission from ref (61). Copyrights 2019 and 2020 American Chemical Society.

Perhaps the best way to begin understanding the structure of the MgCl_2_ melt and in particular the origin of the prepeak is by looking at its crystal structure in Figure 9. Here, we do not mean to imply that the melt is a disordered version of the crystal. Instead, what we claim is that features in the melt are reminiscent of those in the crystal, which can also be described as having Cl^–^-decorated Mg^2+^ networks in which the distance between adjacent cations that are in-network is different from that of adjacent cations that are across networks. The (003) planes shown in Figure 9b are the lowest observed *q* planes, and atoms associated with these planes resemble those associated with the prepeak in the liquid phase (see Figure 9g). These planes go through the Cl-decorated Mg^2+^ charge networks and the distance between planes is the distance between the networks. Notice that there is no sharing of Cl^–^ counterions across networks, only along networks. If we compare the FSDP in the crystal with that of the red line associated with the melt in Figure 9a, it is obvious that the prepeak appears at lower *q* values (longer distance) in the disordered phase; this is to be expected because of the lower density of the liquid phase. In the liquid phase, networks are shorter and randomly distributed and the coordination number of Mg^2+^ is lower than in the crystal, yet for practical purposes the prepeak has a similar origin in both phases. Notice also that Figure 9c,d are instances of what in the previous section we defined as charge alternation behavior (i.e., the typical distance between in-network cations that share counterions like in Figure 9f of the melt). Instead, Figure 9e represents one of the many possible version of what we have called in the past^58,117,124^ adjacency correlations since the distance between planes is associated with the distance between adjacent cations and anions.

### Effects of Temperature and Dilution on the Prepeak

It is intuitive to expect features in *S*(*q*) to become less intense as the temperature increases; this, after all, is typical Debye–Waller behavior. Yet, both for ILs and molten salts the prepeak is special in that one commonly finds non-Debye–Waller behavior; see, for example, ref (188) in the case of ILs and ref (61) in the case of molten salts. In the case of MgCl_2_, two patterns govern the topology of the liquid state. The first pattern is the charge alternation along networks and the second pattern is the typical distance between or across these networks. As temperature increases, the likelyhood of long chains of Mg^2+^ that share Cl^–^ counterions diminishes; this results in the expected Debye–Waller behavior for *S*(*q*) in the charge alternation region around 2 Å^–1^. Concomitant with a decrease in the prevalence of corner- or edge-sharing^189^ Cl^–^-decorated Mg^2+^ networks, the likelihood of finding nearby cations that do not share counterions, associated with the prepeak, becomes larger. At least in the case of MgCl_2_, this statistical effect of fewer in-network interactions and more across-network interactions appears to be the reason for the Debye–Waller behavior of the charge alternation peak and the non-Debye–Waller behavior of the prepeak.

**Figure 10:**
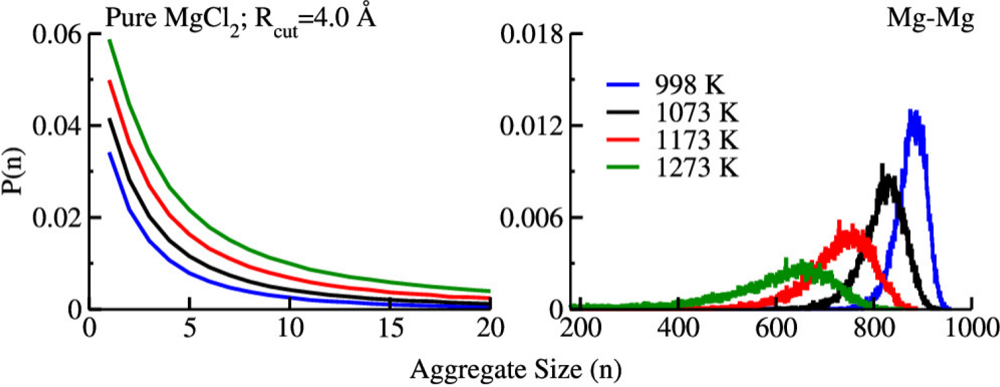
Probability of Cl^–^-decorated Mg^2+^ aggregates as a function of temperature. Reprinted with permission from ref (84). Copyright 2020 American Chemical Society.

**Figure 11:**
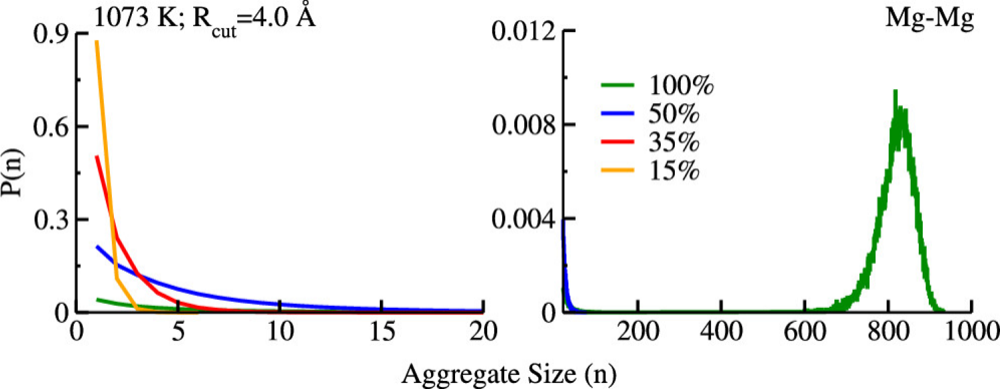
For 1073 K, probability of Cl^–^-decorated Mg^2+^ aggregates as a function of dilution. At 100 mol % MgCl_2_ there is a significant probability of large aggregate sizes, but the probability decreases rapidly upon dilution. Reprinted with permission from ref (84). Copyright 2020 American Chemical Society.

Figure 10 shows the probability of Cl^–^-decorated Mg^2+^ aggregate sizes as a function of temperature; the left panel highlights the smaller networks side of the distribution and the right panel the larger networks. The effect of temperature is as expected from the Debye–Waller behavior of the charge alternation peak in *S*(*q*); larger networks disappear at high *T*. The reader is asked to compare these graphs with those in Figure 11 where the size of Mg^2+^ aggregates is studied as a function of dilution with KCl. We see from Figure 11 that the monovalent salt acts as a powerful Mg^2+^ network disruptor with significant implications on transport properties such as viscosity.

## Conclusions

Ionic liquids and high-temperature inorganic molten salts share many characteristics but are also different in important ways. Molten salts exist in a regime of low viscosities whereas ILs are often much more viscous. From a structural perspective, both are characterized by charge alternation, a feature that always manifests in the partial subcomponents of *S*(*q*) as two peaks and one antipeak. Whether the sum of these components results in a peak in the overal *S*(*q*) depends mostly on contrast from the specific scattering technique. Some ionic liquids and molten salts also display a first sharp diffraction peak. In the case of the most common ILs, this feature is simply due to polarity alternation; this is the pattern of apolar domains separated by charge networks or that of charge networks intercalated by apolar domains. For molten salts, the origin of the prepeak is more complex as it does not trivially arise from an alternation of same-type and opposite-type species. Yet, it would appear that at least in some cases, the separation between charge networks (albeit without a molecular spacer) is also the origin of the prepeak for molten salt systems. *In this case the two typical length scales, associated with the in-network charge alternation peak and the across network prepeak, simply differ because of the way multivalent cations are solvated by counterions*. The charge alternation peak is associated with cations that, because they belong to the same network, necessarily share counterions; the prepeak instead is associated with cations that are also close in distance but are each solvated by a distinct set of anions. These two characteristic distances are difficult if not impossible for nonpolarizable force fields to capture without the use of extraneous terms in the potential. Even polarizable force fields require damping functions in the charge-induced dipole terms to accurately reproduce the two distinct length scales. This brings us to the topic of force field accuracy and transferability, which is much more of a problem for high-temperature molten salts than for ILs. For ILs, features in *S*(*q*) occur at significantly lower *q* values than the same features in the case of molten salts. This means that small inconsistencies across force fields will result in minor changes in *S*(*q*) for ILs but can wreak havoc in the subtle balance of shorter distances that cause the periodicities associated with peaks in *S*(*q*) for molten salts. For molten salts with small multivalent ions and polarizable counterions, the classical point-induced dipole approximation fails and complex corrections are required to reproduce quantum mechanical results and capture features like the prepeak in *S*(*q*). In the case of ILs, polarization is also important, particularly to properly capture transport properties, but structural properties are less sensitive to the details of the force field. This is in part because the distance between ions is larger and also because formal charges are smeared across atoms in the case of ILs. These are in fact some of the features that make salts composed of these larger and softer ions liquid at room temperature.

Whereas measurements on ILs can be difficult due to their hygroscopicity, the same measurements for high-temperature molten salts have the extra complexities associated with impurity reactivity, the need for special setups and the background signal of furnaces among other issues. Furthermore, a significant fraction of these complex measurements have been done in the past using pioneering instruments that among other issues do not go to the large q values that current facilities can achieve. This becomes an issue because often *S*(*q*) is not flat at the cuttoff point and inversion of the data to obtain real space PDFs becomes problematic. The consequence of this is real space data deeply entrenched in the literature, such as coordination numbers for the ions and solvation geometries, that may need to be revisited. This offers tremendous opportunities to impact atomic and quasi-chemical models needed to predict properties, some of them very difficult to measure for molten salt systems.

## Notes

This manuscript has been authored in part by UT-Battelle, LLC, under contract DE-AC05-00OR22725 with the US Department of Energy (DOE). The US government retains and the publisher, by accepting the article for publication, acknowledges that the US government retains a nonexclusive, paid-up, irrevocable, worldwide license to publish or reproduce the published form of this manuscript, or allow others to do so, for US government purposes. DOE will provide public access to these results of federally sponsored research in accordance with the DOE Public Access Plan (http://energy.gov/downloads/doe-public-access-plan).
